# Effect of Different Rootstocks on the Salt Stress Tolerance and Fruit Quality of Grafted Eggplants (*Solanum melongena* L.)

**DOI:** 10.3390/plants12203631

**Published:** 2023-10-20

**Authors:** Maryam Mozafarian, Barbara Hawrylak-Nowak, Noémi Kappel

**Affiliations:** 1 Department of Vegetable and Mushroom Growing, Hungarian University of Agriculture and Life Sciences, Villányi út 29-43, H-1118 Budapest, Hungary; 2 Department of Botany and Plant Physiology, Faculty of Environmental Biology, University of Life Sciences in Lublin, Akademicka 15 st., 20-950 Lublin, Poland; bhawrylak@yahoo.com

**Keywords:** eggplant grafting, salinity, yield, ion accumulation, Na^+^ partitioning

## Abstract

Vegetable grafting is considered a rapid, non-chemical alternative method to relatively slow and expensive breeding to overcome the adverse effect of salinity. Therefore, a soilless experiment was performed to determine the salinity tolerance of eggplant (*Solanum melongena*) cv. Madonna grafted onto two different rootstocks, *Solanum grandifolium* × *Solanum melongena* (SH) and *Solanum torvum* (ST), as well as self-grafted (SG) and self-rooted (SR) as controls. All groups of plants were treated with 0 mM NaCl or 80 mM NaCl. A significant decrease in the relative leaf chlorophyll content (SPAD value) and chlorophyll concentrations were found in response to NaCl. However, the grafted plants had a higher photosynthetic pigment level than the non-grafted plants grown under saline conditions. Grafting eggplants onto SH significantly enhanced the total fruit yield as compared to the self-rooted plants exposed to salinity by increasing the average fruit weight. Moreover, salt stress significantly increased the whitening index and oxidation potential of fruits. The plants grafted onto SH or ST accumulated more Na^+^ in their roots than in their fruit or leaves, thus the Na^+^ partitioning between the above-ground and root parts most probably determines the increased salinity tolerance of the grafted ST and SH plants. To conclude, both the SH and ST rootstocks protected the scions against salinity; the scion showed both increased photosynthetic pigment concentration and chlorophyll fluorescence parameters as well as a lower Na^+^ concentration under stress that resulted in a higher fruit yield and quality.

## 1. Introduction

Horticultural crop production is strongly influenced by fluctuations in environmental factors that have a key impact on plant growth and development. Soil salinization and water irrigation is an important issue in agriculture across the world but mainly in arid and semi-arid regions. Excessive salinity evokes a significant reduction in the area of cultivated land, plant growth, and thus plant productivity [1,2]. Salt stress has various detrimental effects on plants, including a reduction in the photosynthetic rate and chlorophyll content, altering the anatomy and physiology of the plant, increasing reactive oxygen species (ROS) generation, and consequently decreasing plant development and production [3,4]. The accumulation of sodium (Na^+^) and chloride (Cl^−^) in plant tissues causes osmotic stress and interferes with many metabolically crucial compounds and other ions [5].

Eggplant (*Solanum melongena* L.) is a popular and widely cultivated warm-season vegetable crop [6]. Eggplant is moderately sensitive to salt stress (Akinci et al., 2004), with a salinity threshold of 1.5 dS m^−1^ [7]. Previous studies have indicated eggplant’s morphological and biochemical responses to salt stress [8,9,10]. Savvas and Lenz [11] and Abbas et al. [9] showed that NaCl exposition had no effect on the vegetative growth or flower quantity but drastically impaired fruit production and the mean fruit weight of this species.

Several breeding and biotechnology projects have been conducted over the years to generate relatively tolerant crops capable of delivering an economic yield in salt conditions. However, this effort is very challenging due to the genetic complexity of salinity tolerance mechanisms. The grafting of commercial cultivars onto tolerant rootstocks may be a promising tool to alleviate the problems caused by abiotic and biotic stress-related issues [12,13,14]. The rootstock is the primary component that enables the plant to resist stressors, e.g., salinity. Hence, selecting proper rootstocks could enhance scion tolerance by limiting Na and Cl translocation to the shoot, maintaining efficient photosynthesis, and absorbing adequate water and nutrients [15,16,17]. However, information on the yield and quality of the grafted eggplants onto different rootstocks on salinity tolerance is conflicting and still incomplete.

Previously, several studies have been conducted to determine the impact of rootstocks on the plant growth, fruit yield, and quality of eggplants [18,19,20,21,22,23]. According to Wei et al. [24]., grafting eggplant cv. Suqiqie onto salt-resistant rootstock *Solanum torvum* cv. Torvum Vigour results in resistance to salt stress. In turn, Wei et al. [25] demonstrated that seedlings grafted onto *Solanum torvum* were more tolerant to stress than non-grafted plants due to enhanced antioxidant enzyme activity and polyamines accumulation. Earlier studies on grafting eggplant indicated that salt stress damage could be mitigated by increased proline content, enzyme activity, enhanced photosynthesis, increasing N and P level, decreasing the Na^+^/K^+^ value, and by achieving a balanced absorption of Ca, Mg, Cu, Fe, Zn, and Mn [26,27]. Semiz and Suarez [28] conclude that the salt tolerance of grafted eggplant onto Maxifort rootstock is associated with a reduced Na^+^ and increased Ca^2+^ and K^+^ uptake by rootstock. As grafted plants onto *S. torvum* were found to be less sensitive to iodine toxicity, this was more likely a result of the plants being able to withstand higher levels of iodine [29].

Numerous researchers have used *S. torvum,* a wild relative of eggplant, as a rootstock for eggplant exposed to Cd [30], NaCl [31], and a wide variety of soilborne pathogens and nematodes (Gisbert et al., 2011). However, the use of *S. torvum* as a rootstock is limited due to the lack of rapid and homogeneous seed germination even under favorable conditions [19,32]. Therefore, there is a need to distinguish new salinity-resistant alternate rootstocks for eggplant. Another promising rootstock is Taibyou, a Japanese rootstock cultivar developed by Takii Seeds. It is an interspecific hybrid of *Solanum grandifolium* and *Solanum meloengena* resistant to Verticillium wilt and Fusarium wilt. Additionally, Saito et al. [33] and Gao et al. [34] examined Taibyou for resistance to root-knot nematode and cold tolerance.

This research aimed to investigate the physiological responses and Na^+^ and Cl^−^ distribution as well as evaluate the fruit yield and quality of grafted eggplants irrigated with saline water. The hypothesis that grafting eggplants onto different rootstocks increases the plant’s salt resistance and affects the accumulation of inorganic anions in *S*. *melongena* was tested.

## 2. Material and Methods 

### 2.1. Plant Material, Growth Conditions, and Experimental Treatments

The experiment was conducted in an unheated plastic house at the Experimental and Research Farm, Department of Vegetable and Mushroom production, the Hungarian University of Agriculture and Life Sciences, Budapest, Hungary (47°23′49″ N, 19°09′10″ E, 120 m a.s.l.).

Eggplant (*Solanum melongena* L.) cv. Madonna as a scion was purchased from Monsanto Seed Company (USA). Madonna is a vigorous eggplant cultivar with an extensive root system. It yields fruit that is medium dark in color and weighs between 350 and 400 g. The pulp of the fruit is white and contains a few seeds. It is particularly well-suited for greenhouse cultivation. In turn, Taibyou is an interspecific hybrid of *Solanum grandifolium* × *Solanum melongena* which was purchased from Takii Seeds Company (Japan). Torvum (*Solanum torvum)* is one of the most commonly used rootstocks for eggplants due to its high vigor and complete graft compatibility with eggplant scions. It was previously demonstrated that 80 and 90 mM NaCl concentrations were critical for inhibition of eggplant growth [5,35]. According to these findings, we exposed the eggplant to 80 mM NaCl in our experiments.

The research was carried out as a factorial experiment using a completely randomized design (CRD) to determine the influence of different rootstocks on salt damage mitigation. The rootstocks used included the following: Taibyou cv. *Solanum grandifolium* × *Solanum melongena* (SH), *Solanum torvum* (ST), self-grafted *Solanum melongena* (SG), and self-rooted *Solanum melongena* (SR). The NaCl treatment included 80 mM NaCl (salt stress) and 0 mM NaCl (as control) with four replications and five plants in each replication. The eggplant cv. Madonna was used as a scion. All the rootstock seeds (SH and ST) were sown ten days before eggplant cv. Madonna in seedling trays and exposed to 18 h of light, a temperature of 25–26 °C, and a humidity of 70–80%. Eggplant seedlings (with an appropriate diameter and 4–5 leaves) were grafted onto ST and SH rootstocks and for self-grafted seedlings (Madonna were grafted onto Madonna) using the cleft grafting method and acclimatized to natural conditions in high humidity and low light after one week. Non-grafted Madonna seedlings were used as self-rooted plants, without grafting. Three weeks after grafting, all plants were transplanted into 10 L pots containing peat substrate and placed in an unheated plastic house. All phytotechnical work for eggplant cultivation in the greenhouse was completed consistently. Daily irrigation was performed with normal water and commercial fertilizer solution was applied twice a week. Each plant received 1 L of water (or nutrient) per day.

Two types of plant nutrient solutions were used: Ferticare I (14:11:25 + microelements) with an EC (electrical conductivity) value of 3.80 dS m^−1^ for the control plants or Ferticare I + 80 mM NaCl with an EC value of 11.40 dS m^−1^ for salt stressed plants. The EC of the water and each fertilizer solution was measured with a manual electrical conductivity meter (HI 98,311 DiST^®^ 5 EC/TDS (total dissolved solids)-Tester). Each plant was irrigated with the same type and amount of nutrient solution based on the established experimental series.

The salinity levels were reached gradually for 6 days by adding the appropriate amount of NaCl to the control solution. The pH and the EC were controlled during each irrigation according to the values measured in the drainage water.

### 2.2. Measurements of Physiological Parameters

- The relative chlorophyll content was measured with a portable chlorophyll meter (SPAD 502; Minolta Camera, Osaka, Japan).
- Leaf discs from the fully developed sixth leaf from the top of three plants per replication were used to determine the photosynthetic pigments. The chlorophyll was extracted from the tissue by grinding with a mortar and pestle in ammoniacal acetone (10 mL).
  The pigment concentrations were determined by spectrophotometry (CT 200 spectrophotometer, Inesa, Shanghai China). The absorbance was measured at 470, 647, and 664 nm. Strain and Svec [36] described the following formulae and extinction coefficients to determine the chlorophyll and carotenoids:

Chlorophyll a: (12.7 × A_664_) − (2.69 × A_647_)

Chlorophyll b: (22.9 × A_647_) − (2.69 × A_664_)

Carotenoids: (1000 × A_470_) − (1.8 × Chl a) − (85.02 × Chl b))/198

- The chlorophyll fluorescence was determined by a portable chlorophyll fluorometer (Model OS5P, Optisciences Inc., Hudson, NY, USA). Before the measurements, the leaves were dark-adapted for 30 min to obtain the Fv/Fm value (Fv and Fm represent the variable and maximum fluorescence, respectively). The quantum yield efficiency of PSII of the light-adapted leaves (Φ_PSII_) was determined using the formula Φ_PSII_ = (Fm′ − Fs) × Fm′, where Fm′ and Fs denote the maximum and steady-state fluorescence yields, respectively, of light-adapted leaves Fs was determined using an actinic light pulse (270 mol m^–2^ s^–1^). At the same time, Fm′ was produced using a 1 s pulse of saturating white light.
- The individual leaves were separated from the stem and weighed to determine their fresh weight (FW). Then, leaves were floated in distilled water inside a closed tube to calculate the turgid weight (TW). After 12 h the leaf samples were weighed. After the imbibition phase, leaf samples were dried in a preheated oven at 80 °C for 48 h to determine their dry weight (DW).
  The RWC (relative water content) was calculated using the following formula [37]:

RWC (%) = ((FW − DW)/(TW − DW)) × 100

- The total leaf water potential (Ψw) was measured with a potentiometer (WP4, Decagon Devices, Washington DC, USA) at the vegetative and reproductive periods.

### 2.3. Fruit Yield and Quality Measurement

Mature fruits were picked weekly, depending on the fruit’s size, color, and glossiness from June to September (15 times).

- Immediately after picking, the weight of each fruit was recorded using a precision balance (EMS, KERN & SOHN, Balingen, Germany) and marketable and unmarketable fruits were identified. The width and length of the fruits were measured with a ruler.
- The firmness of the fruits was determined using a hand-held penetrometer (Insize 9643-4 Fruit Hardness Tester, Insize, Suzhou, China). The pressure value was expressed in kg/cm^3^.
- A pH meter (Hanna HI 98128, Hanna Instruments, Smithfield, VA, USA) was used to measure the acidity of the two sides of each fruit’s flesh in all combinations. The mixtures of three fruit pulps from each replication were blended using a standard blender.
- The total sugar content (TSS%) was measured using the refractometer (PAL-1 Brix 0–53% Digital Hand, Atago, Tokyo, Japan) with the fruit juices.
- The skin color on two sides of three fruits from each replication was determined using a colorimeter (Minolta Chroma CR-400, Minolta Corporation Ltd., Osaka, Japan). The chromaticity of fruits was quantified using L*, a*, and b* color space coordinates which represent the lightness (0 being black and 100 being white), redness [green (−) to red (+)] and yellowness [blue (−) to yellow (+)], respectively. The chroma (C*), hue angles (H°), and color index of red grape (CIRG) were calculated as the following formula:

C* = (a*^2^ + b*^2^)^1/2^, H° = tan^−1^(b*/a*)

CIRG = (180 − H°)/(L* + C*)

- The whitening index (DW), oxidation potential (OP), and color differences (CD) of the fruit pulp were all determined using the same colorimeter. Two fruits from each replication were cut longitudinally with a knife with a straight edge. In the center and lateral zones, the pulp color was measured immediately after cutting (*L*_0_) and 30 min later (*L*_30_). The DW was calculated using the formula [38] as the Euclidean distance between the color coordinates and the pure white coordinates (L* = 100 a* = 0 *b* = 0): DW = ((100 − *L*)_2_ + a^*^_2_ + b^*^_2_)^0.5^.
  The OP of fruits was estimated by L*a*b* values using the following formula [39]:

OP = L*_30_ – L*_0_

Additionally, the CD was calculated as the distance between the color coordinates at 0 and 30 min in Euclidean space: CD = [(L^*^_30_ – L^*^_0_) + (a^*^_30_ – a^*^_0_) + (b^*^_30_ – b^*^_0_).

### 2.4. Determination of Na^+^ and Cl^−^ in Roots, Leaves and Fruits

- The Na^+^ and Cl^−^ concentration in leaves, roots, and fruits tissues was evaluated using an ICP-OES spectrometer (IRIS Thermo Jarrel ASH Corp., Franklin, MA, USA). The homogenous plants samples were used for these analyses [40].

### 2.5. Data Analysis

The experiment was designed as a factorial design with four replications and five plants in each replication. Statistix 8 software (Tallahassee, FL, USA) was used to conduct a statistical analysis of the data. A two-way analysis of variance was used to analyze the data, and means were separated using the Tukey’s multiple range test at a significance level of α = 0.05. Normality was checked by Kolmogorov–Smirnov and Shapiro–Wilk tests. The homogeneity of variances was measured with Levene’s test.

## 3. Results

### 3.1. Effect of Grafting and Salinity on Plant Physiological Traits

The SPAD values decreased significantly (11% compared to the control) in response to salt stress, whereas grafting onto SH enhanced the SPAD value in both the saline and normal conditions (Table 1). The interaction between the salinity level and rootstock type considerably affected the photosynthetic pigments concentration. A significant reduction in the chlorophyll a and b levels were found in the plants exposed to 80 mM NaCl. Despite this, the rootstocks SH, ST, and SG were less damaged than SR. The maximum total chlorophyll concentrations were shown in non-saline conditions in the plants grafted on SH. In turn, in saline conditions, the plants grafted onto SH followed ST, and then SG exhibited less total chlorophyll loss than the SR plants. The interaction effect of the grafting and salinity level revealed that salt stress greatly reduced the carotenoid concentration in SR plants compared to the SR plants grown in non-saline conditions. On the other hand, under salt stress, the carotenoid levels significantly increased in grafted plants (SG, ST, and SH) compared to SR plants in vegetative but not in the generative stage (Table 1).

The interaction effect of the rootstock types and salinity level on the selected chlorophyll fluorescence parameters (Table 2) shows that excessive salinity significantly decreased the Fv/Fm value of eggplant. In contrast, the decrease in this parameter in the grafted plants (SG, ST, and SH rootstocks) was slighter than in the SR plants. Similarly, the Φ_PSII_ value had a greater reduction in the SR plants exposed to NaCl, whereas the highest Φ_PSII_ was observed in the SH × control and SH × salinity treatments.

The salinity slightly decreased the leaf RWC and grafting onto SH and ST improved it; however, these differences were not statistically significant (Table 2). Salt application significantly reduced the leaf water potential in eggplant leaves. Interaction effect of rootstocks type and NaCl treatment showed that grafting on ST and SH induced the decrease in the leaf water potential in salt stress conditions.

### 3.2. Effect of Grafting and Salinity on Fruit Yield and Quality

**Figure 1:**
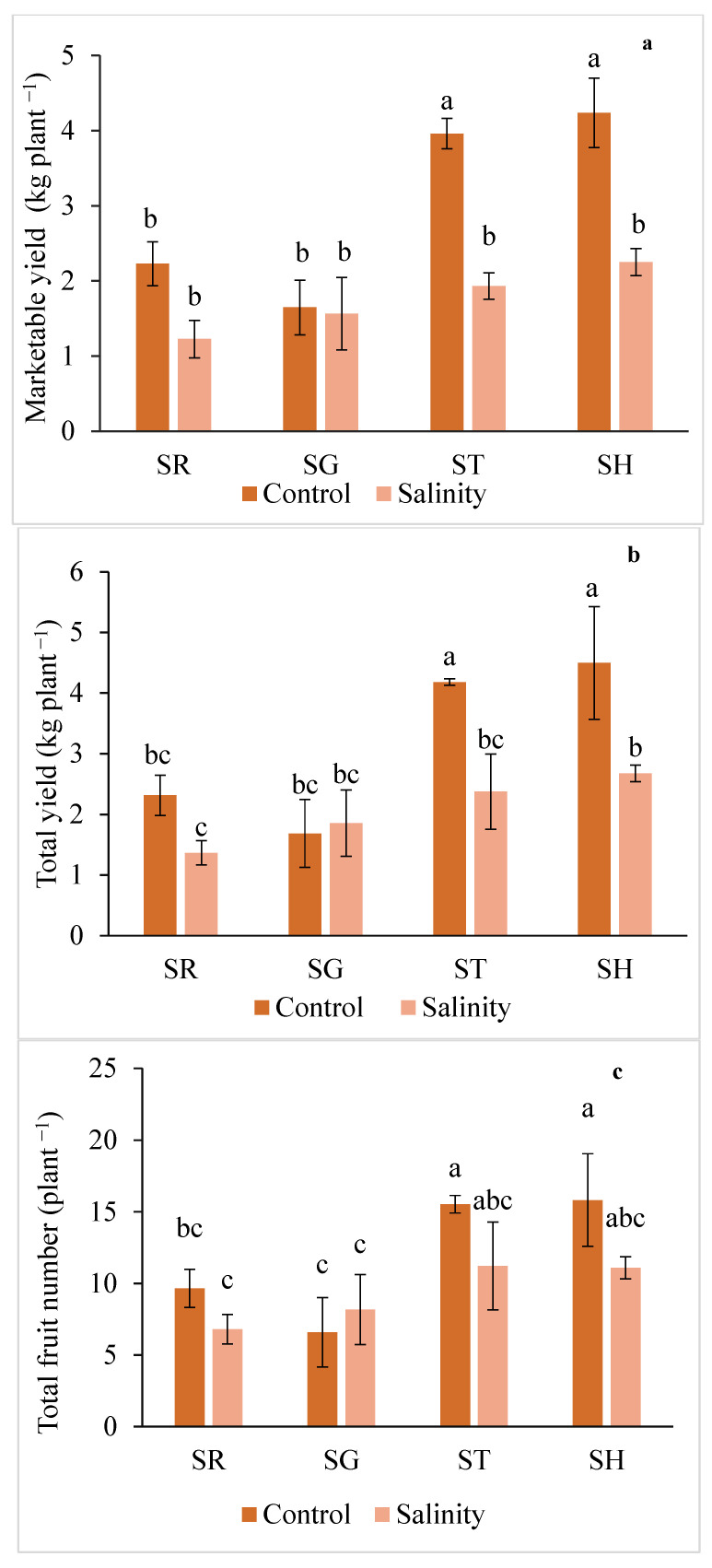

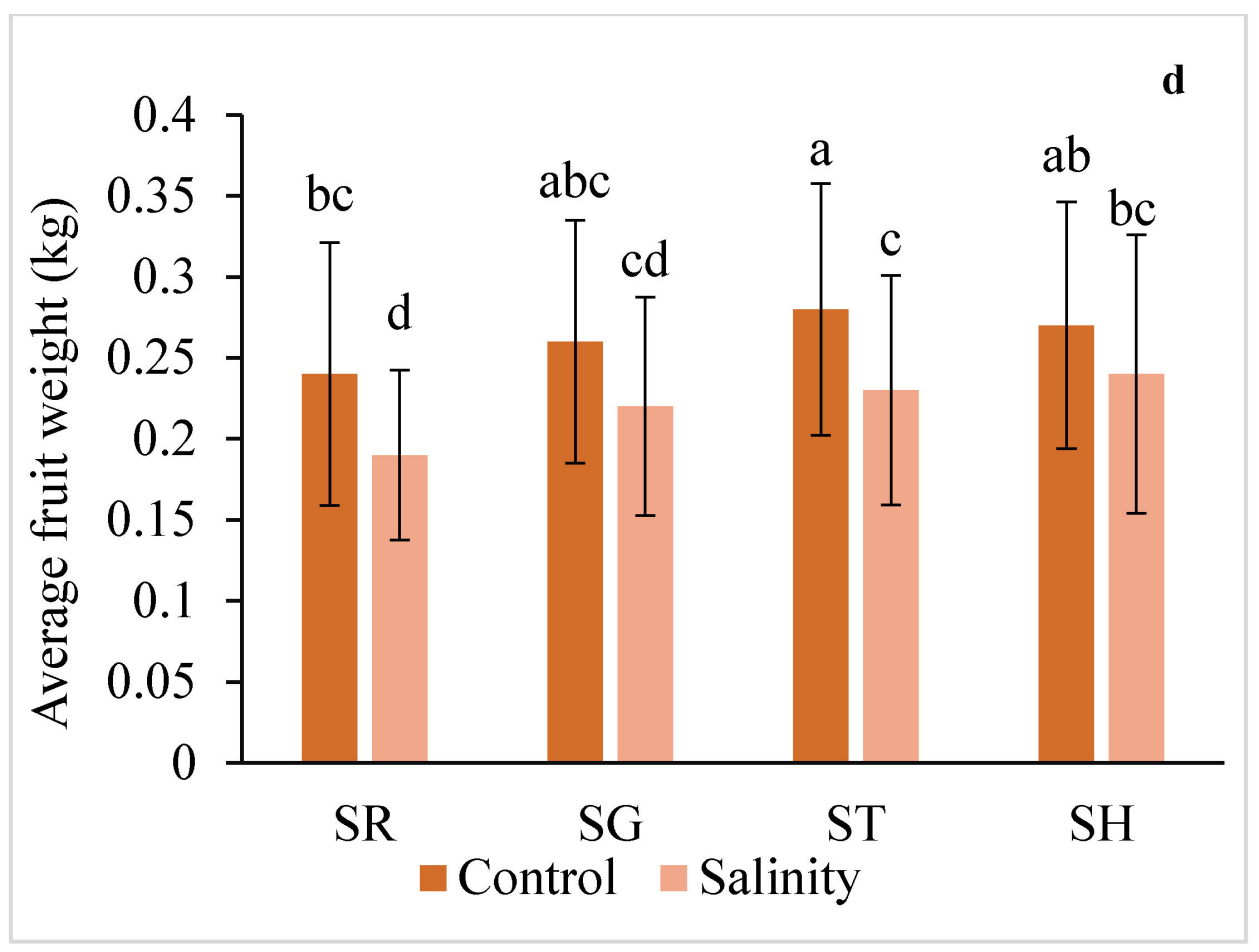
Interaction effect of salinity and use of different rootstocks on total marketable yield (**a**), total yield (**b**), fruit number (**c**), fruit average weight (**d**) of eggplant. Mean values (standard deviation) marked with different letters are significantly different according to Tukey’s multiple range test at *p* < 0.05. SR = self-rooted; SG = self-grafted; ST = *Solanum torvum,* SH = *Solanum grandifolium* × *Solanum melongena* rootstock.

As shown in Figure 1a, the highest total marketable yield was observed in the control × SH and control × ST combinations (4.24 and 3.96 kg plant^−1^, respectively); thus it was much higher compared to the SR plants in the non-stress conditions. A slight decrease in the total yield per plant (Figure 1b) was observed in the SR in response to the salinity treatment; however, grafting onto SH significantly increased the total yield by 20% compared to the 80 mM NaCl stressed SR plants. In the non-stress conditions, the total fruit number per plant (Figure 1c) considerably increased by grafting the plants onto SH and ST. On the other hand, in the salt-stress conditions, this increase was not statistically significant. Moreover, the salinity significantly (by 12%) decreased the average fruit weight compared to the control plants. However, grafting onto ST and SH significantly enhanced the average fruit weight in saline conditions (by 21% and 26%, respectively), in comparison with the non-grafted plants (Figure 1d).

**Figure 2:**
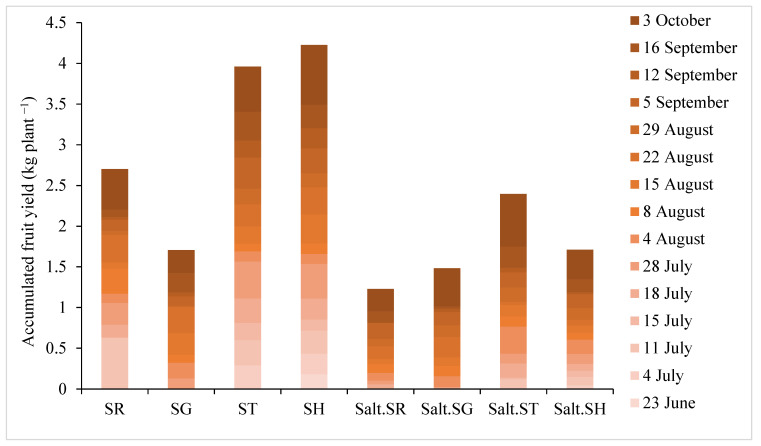
Effect of salinity and use of different rootstocks on accumulated fruit yield per plant during the 15 harvesting times of eggplant. SR = self-rooted; SG = self-grafted; ST = *S. torvum* rootstock, SH = *S. grandifolium* × *S. melongena* rootstock.

The first and second yield of the fruits was obtained from the plants grafted onto SH in non-saline conditions on 28th of June and then from the grafted plants onto SH and ST under both saline and non-saline conditions on 4th of July. Under salt stress, fruits from plants grafted on ST or SH was ready to pick earlier than from SR and SG individuals (Figure 2). Under normal conditions, the number of harvesting fruits and yield per harvest were greater in plants grafted onto SH and ST than in SR and SG.

Salt exposition considerably decreased the length of the fruits. However, this reduction was less in the fruit harvested from the plants grafted onto SH and ST (Table 3). The salinity also significantly decreased the eggplant fruit width, regardless of the rootstock type. The fruits picked from the plants grafted onto SH had the lowest firmness. In turn, the total soluble solids value increased significantly under salinity in the plants grafted onto ST and in SR individuals. On the other hand, grafting, salinity, and their interaction had no effect on the pH value of the fruit tissues (Table 3).

The salinity significantly decreased the L* value of the skin. Grafting of the eggplants onto ST significantly increased the skin a* and chroma values in the saline stress conditions. Moreover, the interaction between the salinity level and rootstock type showed that grafting the eggplant on ST rootstock significantly decreased the CIRG index compared to SR individuals growing in the saline conditions (Table 4).

As shown in Table 5, the salinity significantly decreased the L*_0_ of fruit pulp (by 2.84%). Moreover, grafting the eggplants onto ST and SH decreased the L*_0_ in comparison with the self-rooted plants. The interaction results showed that the lowest L*_0_ was obtained in SH and SG in stress conditions. The DW value ranged from 27.99 (control × SR) to 35.63 (salinity × SH) (Table 5). In turn, the oxidation activity of the fruit pulp was higher in the harvested fruits of the plants grown in saline conditions. Our results showed that the brighter fruits (SR × control; SG × control) had a lower value of chroma, degree of whiteness, and oxidation potential than the other combinations in the stress or non-stress conditions. The interaction effect of the rootstock type and salinity level showed a high OP value in the fruit of SR under stress conditions (about two times more than the SR plant in normal conditions).

### 3.3. Effect of Grafting and Salinity on Na^+^ and Cl^−^ Concentrations

**Figure 3:**
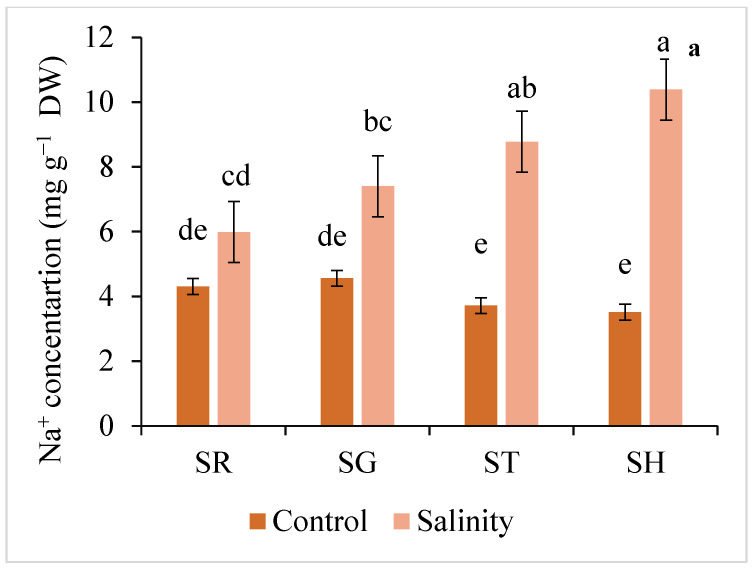

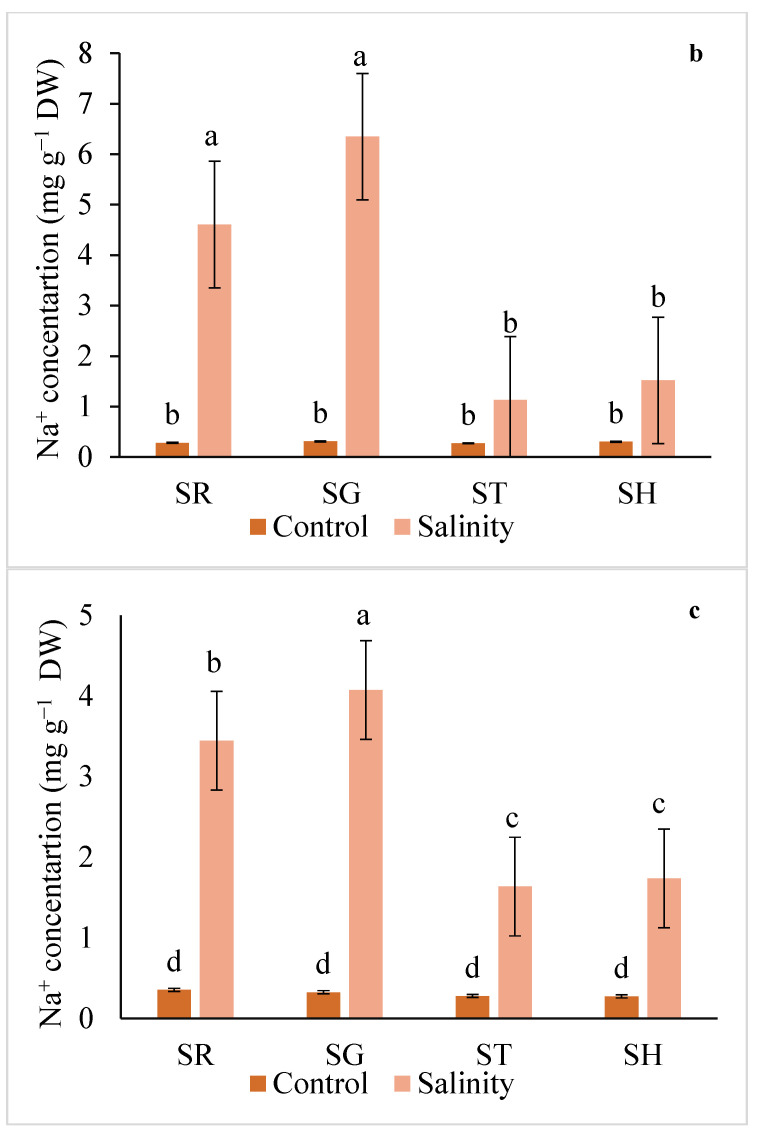
Interaction effect of salinity and use of different rootstocks on Na^+^ concentration in root (**a**), leaf (**b**), and fruit (**c**) of eggplants. Mean values (standard deviation) marked with different letters are significantly different according to Tukey’s multiple range test at *p* < 0.05. SR = self-rooted; SG = self-grafted; ST = *Solanum torvum*; SH = *Solanum grandifolium* × *Solanum melongena* rootstock.

The plants grafted onto the ST or SH rootstocks had a higher Na^+^ concentration in the roots but less in the leaves and fruits than the SR or SG individuals (Figure 3). However, no significant differences were found between SH and ST in the Na^+^ accumulation. The plants grafted onto ST and SH accumulated 14% and 17% more Na^+^ in their roots, respectively, than the SR plants grown in the saline conditions. The highest Na^+^ concentration both in the leaf and fruit was determined in SG plants (Figure 3).

**Figure 4:**
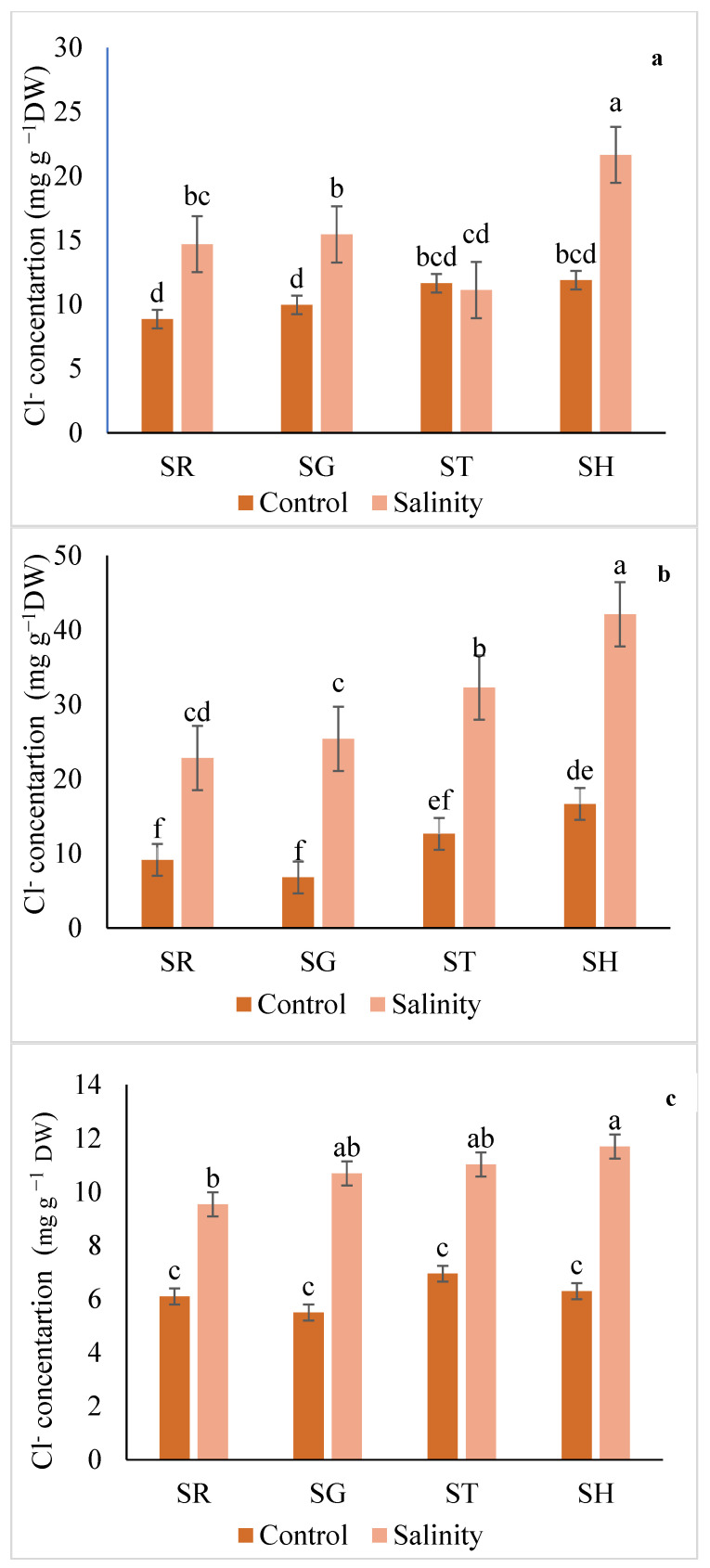
Interaction effect of salinity and use of different rootstocks on Cl^−^ concentration in root (**a**), leaf (**b**), and fruit (**c**) of eggplants. Mean values (standard deviation) marked with different letters are significantly different according to Tukey’s multiple range test at *p* < 0.05. SR = self-rooted; SG = self-grafted; ST = *Solanum torvum*; SH = *Solanum grandifolium* × *Solanum melongena* rootstock.

In general, there was an increase in the Cl^−^ concentrations in the leaves, roots, and fruits of all the grafted combinations compared to the non-grafted plants when subjected to saline conditions (Figure 4). While there was no significant difference in the fruit Cl^−^ concentration between the SG, SH, and ST plants, the level of this element in the leaves and roots of the eggplants was significantly higher in SH than in the other experimental series under salt exposition. In saline conditions, the plants grafted on SH accumulated about 18.5% more Cl^−^ in their fruits than the SR individuals (Figure 4).

## 4. Discussion

The climate change-induced increase in salinity is considered as one of the main stress factors which affect horticultural crops. This phenomenon necessitates the search for a change in the cultivation methods. The use of grafted vegetables can help to mitigate its negative effects, and both the scion and rootstock features can affect the tolerance of grafted plants [12,41]. We found that salinity had a detrimental effect on the eggplants’ physiological traits, yield, and fruit quality. In turn, the grafting of eggplants onto SH or ST rootstocks minimized this effect. For example, under salt stress, the total chlorophyll content in the eggplant scions grafted onto ST at the generative stage was about 40% higher than in non-grafted plants. In line with our results, Colla et al. [42,43] and Hegazi et al. [44] reported that the grafted plants exposed to salt stress had a higher SPAD index and chlorophyll content than nongrafted plants or self-grafted plants. Some results indicate that grafting could improve the adverse impact of salinity by preventing chlorophyll degradation [39,45].

One evaluation method of plants’ response to stress conditions is monitoring of the chlorophyll fluorescence parameters. In our study, the inhibitory effect of NaCl on F_v_/F_m_ values in grafted plants was much milder than the non-grafted or self-grafted plants. Moreover, the Φ_PSII_ was more sensitive to salt stress than the F_v_/F_m_ parameter. In line with our results, the values of the F_v_/F_m_ in the self-rooted and self-grafted plants were lower than in the grafted tomato and cucumber under NaCl stress [39,46,47]. These findings show that grafting may be able to prolong photoinhibition under salt exposition. Moreover, it was found that the higher Φ_PSII_ values in the grafted plants were positively linked to better photosynthesis efficiency under salt stress and Φ_PSII_ was significantly lower with minor or no decline in the F_v_/F_m_, which might be attributed to more energy being wasted thermally in Φ_PSII_ [46].

Under salinity, the water uptake by plants decreases due to lowering the water potential of the soil solution, which induces physiological drought [48]. Orsini et al. [49] found that the leaf water potential decreased under salinity (40 and 80 mM NaCl), and this decrease was higher in non-grafted than self-grafted melon plants. In turn, Santa-Cruz et al. [50] observed an increase in the leaf water content in grafted tomato in saline conditions, which could be attributed to some changes in the root characteristics. Also, in our study, salinity significantly decreased the leaf water potential, but grafting onto different rootstocks improved this parameter. A similar finding to our result was reported by Sanwal et al. [51], who reported that the grafted plants showed a higher RWC under stress, suggesting that the rootstocks maintained the uptake of water under salt stress.

In non-stress conditions, increasing the total fruit yield of the grafted eggplant has been reported in several studies, depending on the scion and rootstock cultivars and their compatibility [18,19,20,23]. In the present study’s experiments, a significant increase in the total and marketable fruit yield of the grafted plants grown was found in the control but not in the salt stress conditions. This increase was more dependent on the total fruit number per plant than on the average fruit weight. Only the SH individuals were characterized by a higher yield under salinity compared to the self-rooted plants. Moreover, the ST and SH plants had a higher average fruit weight than the SR individuals. The higher fruit yield of the grafted plants relative to the self-rooted plants was reported in eggplants, tomatoes, and peppers grown in saline conditions [17,24,28,45,50,52]. The higher yield of grafted plants under salinity is probably related to a lower accumulation of Na^+^ and Cl^−^ in tissues. Also, it can be attributed to an increased nutrient uptake, enhanced production of endogenous hormones, improved antioxidant activity, and thus enhanced the scion vigor [12,39,53].

Salt tolerance is primarily due to the ability of plants to inhibit the translocation of ions between the roots and shoots, which has been associated with lower ionic ratios in the shoots of grafted vegetables [51]. In our study, grafting differentially affected the Na^+^ and Cl^−^ bioconcentration in individual salt-stressed plant parts. While Na^+^ accumulation increased in the roots of the ST and SH plants in comparison to the SR or SG individuals, it significantly decreased in the fruits and leaves. Meanwhile, the Cl^−^ concentration showed a different trend; the level of this anion significantly raised in the leaves of ST and SH plants and in the fruits and roots of SH plants in comparison to the SR individuals. These relationships indicate that the reduced Na^+^ translocation to the above-ground parts may mainly determine the increase in the salinity tolerance of the grafted ST and SH plants. Similarly, grafting decreased the concentrations of Na^+^, but not Cl^−^ in the leaves of melon and watermelon [12]. In turn, the increased resistance to salt stress of eggplant cv. Angela grafted onto tomato Maxifort rootstock was attributed to a reduced uptake of Na^+^ and increased uptake of Ca^2+^ and K^+^ by rootstock [28]. Recently, Sanwal et al. [51] showed that salt-resistant eggplant rootstocks may affect Na^+^ partitioning in tomato scions by promoting a higher Na^+^ accumulation in the older leaves and lower accumulation of this ion in the younger leaves of scions, thus enhancing the tolerance of tomato to salinity. In turn, some reports indicate that the use of tolerant rootstocks can increase the salt tolerance of scions by reducing both the Na^+^ and Cl^−^ translocation to the shoots [12,13,54]. Moreover, Estañ et al. [50] indicated that the varied marketable yield responses were attributed to the diverse abilities of the rootstocks to regulate the transport of toxic salt ions. Contrary to our results, tomatoes grafted onto ‘Unifort’ rootstock under saline circumstances accumulated significantly less Cl^−^ than non-grafted plants [55]. In turn, Santa-Cruz et al. [50] found that the accumulation of Na^+^ and Cl^−^ in tomato leaves depended on the graft combination. In comparison to self-grafted ‘Moneymaker’ plants, the combination of ‘Moneymaker’ and ‘Kyndia’ had a lower Na^+^ bioconcentration and comparable Cl^−^ content. However, this response was different for the combination of ‘UC-82 B’ and ‘Kyndia,’ in which a decreased accumulation of both Na^+^ and Cl^−^ was noted.

Depending on the rootstocks and salinity levels, the TSS can either increase or decrease. While Rouphael et al. [56] and Turhan et al. [57] concluded that grafted tomato plants have a lower TSS content than non-grafted plants, Savvas et al. [58] and Di Gioia et al. [59] reported no effect of grafting combinations on the TSS content. Those plants that were grafted onto ST under saline conditions showed the highest TSS. In line with our results, Sanwal et al. [51] reported that the fruits from grafted plants treated with saline irrigation had a higher TSS. Among the analyzed color parameters of the full ripe eggplant fruits, the CIRG index increased with salinity and grafting in our study. Kacjan Maršić et al. [60] reported the darkness of the fruit of eggplant cv. Epic decreased in self-grafted plants in comparison with plants grafted onto rootstock cv. Beaufort. They concluded that the CIRG and browning indexes were highly dependent on the combination of the grafting and scion. Also, the concentration of anthocyanins, as the essential compounds for eggplant peel colors, depended on grafting [20,60]. The reason for this could be that the appropriate nitrogen level that is required for developing the red color in eggplant skin may decrease under salt stress conditions. At the same time, grafting can enhance the accumulation of this macronutrient [61]. Moreover, both consumers and industry prefer white-fleshed and late browning eggplant varieties. It was found that grafting onto *Solanum torvum* showed no discernible effect on the pulp lightness, whereas the fruits grafted onto *Solanum paniculatum* or *Solanum macrocarpon* affected the pulp lightness negatively [20]. Moncada et al. [18] grafted four eggplant cultivars onto *Solanum torvum* and they reported that grafting did not affect the fruit’s lightness or its browning potential. The flesh and skin color appear to be determined mainly by the cultivar and rootstock combinations [18]. In turn, our results indicated that the fruits harvested from SH-grafted plants had the highest whitening index (DW).

A comparison of landraces, commercial, and hybrid eggplant varieties revealed that landrace varieties produced fruit closer to pure white (DW) than commercial varieties. In contrast, hybrid and commercial varieties produced fruit with a lower mean degree of browning after exposure to air (OP and CD parameters) than landrace varieties. This phenomenon is probably due to the selection of breeding programs [37]. The oxidation potential of the phenolics in the fruit flesh causes it to brown when exposed to air, decreasing the apparent quality. Polyphenol oxidases display a wide range of activity among eggplant varieties [62], which can be a reason for less browning (OP) in self-rooted plants than grafted onto SH or ST rootstocks. On the other hand, as browning is correlated to phenolic compound level and the salinity increased the total phenols in eggplant fruits [44] it can be stated that salinity can increase the browning index of fruits.

## 5. Conclusions

The results indicate that use of both rootstocks (*Solanum grandifolium* × *Solanum melongena* and *Solanum torvum*) are able to improve the tolerance of eggplant scions (*Solanum melongena*) cv. Madonna to salinity stress. The increased tolerance of the grafted plants to salinity in terms of the fruit yield was associated with reduced Na^+^ in the shoots and fruits, a higher maximal quantum efficiency of photosystem PSII, and an increased chlorophyll concentration and water content. We found that grafting onto SH has almost a similar effectivity to ST under salt stress and can be used as a salt-resistant rootstock. Based on our results, we suggest that future studies and efforts for improvement of the salt tolerance of eggplants by grafting using appropriate rootstocks should be focused on improving the Na^+^ partitioning and/or exclusion mechanisms.

## Figures and Tables

**Table 1:** Effect of rootstocks type on SPAD value and photosynthetic pigments concentration of eggplants grown in control or salt stress conditions.

Vegetative Stage						
Salinity	Rootstocks	SPAD	Chlorophyll *a* (mg g^−1^ FW)	Chlorophyll *b* (mg g^−1^ FW)	Total Chlorophyll (mg g^−1^ FW)	Carotenoids (mg g^−1^ FW)
Control	SR	54.30 b,c	18.03 ± 1.91 b,c	3.07 ± 0.58 b,c	21.10 ± 2.67 b,c	1.43 ± 0.14 a,b
	SG	52.55 b–d	16.90 ± 2.17 b,c	3.12 ± 0.57 b,c	20.03 ± 2.68 c	1.51 ± 0.41 a,b
	ST	55.27 ab	18.49 ± 0.81 b	3.44 ± 0.78 a,b	22.36 ± 1.11 b	1.80 ± 0.49 a
	SH	58.22 a	23.46 ± 2.69 a	3.87 ± 0.47 a	26.90 ± 3.46 a	1.58 ± 0.34 a,b
Salinity	SR	48.45 e	9.76 ± 2.54 e	1.68 ± 0.91 e	11.44 ± 3.26 e	0.41 ± 0.60 c
	SG	50.60 d,e	14.55 ± 1.96 d	2.44 ± 0.60 d	17.07 ± 2.55 d	1.31 ± 0.09 b
	ST	51.35 c–e	14.79 ± 1.33 d	2.61 ± 1.25 c,d	17.23 ± 2.31 d	1.34 ± 0.73 b
	SH	52.30 b–d	16.35 ± 1.35 c,d	2.74 ± 1.36 c,d	19.10 ± 2.00 c	1.33 ± 0.48 b
Main effects						
Control		53.86 a	19.08 a	3.28 a	22.36 a	1.56 a
Salinity		51.90 b	14.00 b	2.46 b	16.47 b	1.12 b
	SR	51.82 b,c	16.41 b	2.21 c	19.17 b	1.10 b
	SG	49.52 c	13.05 c	2.75 b	15.27 c	1.33 a,b
	ST	53.91 a,b	16.52 b	3.24 a	19.76 b	1.51 a
	SH	56.26 a	20.18 a	3.28 a	23.46 a	1.41 a
Analysis of variance						
Salinity		*	***	***	***	***
Rootstock		***	**	***	***	*
Salinity× Rootstock		*	**	*	*	***
CV		4.56	8.26	13.98	7.85	20.25
Generative stage						
Control	SR		25.68 ± 2.99 b–d	5.15 ± 0.65 a–c	30.65 ± 3.49 c,d	1.73 ± 0.70 c
	SG		26.95 ± 2.40 b,c	5.56 ± 2.28 a,b	32.10 ± 4.41 b,c	2.08 ± 2.15 b,c
	ST		31.48 ± 6.04 a	6.15 ± 7.19 a	37.64 ± 17.90 a	2.49 ± 1.98 a,b
	SH		28.88 ± 7.64 a,b	5.68 ± 1.86 a,b	34.56 ± 6.08 a,b	2.67 ± 0.79 a
Salinity	SR		17.20 ± 10.72 f	3.73 ± 3.34 d	20.94 ± 12.95 f	1.16 ± 0.79 d
	SG		21.47 ± 4.70 e	4.30 ± 3.40 c,d	26.19 ± 7.03 e	1.54 ± 0.62 c,d
	ST		25.09 ± 6.30 c,d	4.71 ± 2.03 b–d	29.39 ± 7.32 c–e	1.55 ± 0.67 c,d
	SH		22.66 ± 3.45 d,e	4.91 ± 2.26 b,c	28.23 ± 2.42 d,e	1.68 ± 0.95 c,d
Main effects						
Control			28.25 a	5.47 a	33.77 a	2.24 a
Salinity			21.60 b	4.58 b	26.18 b	1.48 b
	SR		21.44 b	4.32 c	25.77 b	1.62 b
	SG		26.02 a	4.72 b,c	30.37 a	1.64 b
	ST		27.07 a	5.86 a	32.93 a	2.02 a,b
	SH		25.07 a	5.19 ab	30.74 a	2.17 a
Analysis of variance						
Salinity			***	**	***	***
Rootstock			***	**	***	**
Salinity × Rootstock			*	*	*	*
CV			9.12	14.56	8.36	21.08

Different letters within the same column indicate that means ±standard deviation (four replications and 5 plants in each replication) are significantly different according to Tukey’s multiple range test at: *p* < 0.05 (*), *p* < 0.001 (**), or *p* < 0.0001 (***). SR = self-rooted; SG = self-grafted; ST = *Solanum torvum,* SH = *Solanum grandifolium* × *Solanum melongena.*

**Table 2:** Effect of rootstocks type on chlorophyll fluorescence parameters and leaf water potential in eggplants grown in control or salt stress conditions.

Salinity	Rootstocks	Fv/Fm	Φ_PSII_	Leaf Water Potential (Ψw MPa)	RWC (%)
Control	SR	0.86 ± 0.56 a	0.80 ± 0.01 a	−2.58 ± 2.19 a	76.89 ± 11.37 b,c
	SG	0.86 ± 0.01 a	0.80 ± 0.02 a	−1.81 ± 1.56 a	78.55 ± 4.45 b,c
	ST	0.86 ± 0.02 a	0.81 ± 0.04 a	−1.91 ± 1.37a	91.42 ± 4.64 a
	SH	0.87 ± 0.02 a	0.81 ± 0.02 a	−1.76 ± 1.51a	87.57 ± 15.52 a,b
Salinity	SR	0.80 ± 0.01 d	0.65 ± 0.03 d	−6.00 ± 0.65 b	69.25 ± 7.06 c
	SG	0.82 ± 0.05 c,d	0.69 ± 0.01 c	−4.02 ± 3.18 a,b	72.16 ± 3.95 c
	ST	0.83 ± 0.01 b,c	0.72 ± 0.01 b,c	−3.32 ± 0.44 a,b	75.18 ± 8.72 b,c
	SH	0.85 ± 0.03 a,b	0.73 ± 0.01 b	−2.82 ± 1.30 a,b	75.92 ± 26.45 b,c
Main effects					
Control		0.86 a	0.80 a	−2.01 a	83.61 a
Salinity		0.82 b	0.70 b	−4.04 b	73.13 b
	SR	0.83 b	0.72 b	−2.95	76.03
	SG	0.84 ab	0.75 a	−2.31	77.23
	ST	0.84 ab	0.77 a	−3.96	78.41
	SH	0.86 b	0.76 a	−2.89	81.79
Analysis of variance					
Salinity		***	***	***	***
Rootstock		*	***	***	ns
Salinity× Rootstock		*	**	***	***
CV		2.78	3.73	10.81	6.72

Different letters within the same column indicate that means ±standard deviation (four replications and 5 plants in each replication) are significantly different according to Tukey’s multiple range test at: *p* < 0.05 (*), *p* < 0.001 (**), or *p* < 0.0001 (***), ns = non-significant. SR = self-rooted; SG = self-grafted; ST = *Solanum torvum* rootstock, SH = *Solanum grandifolium* × *Solanum melongena* rootstock.

**Table 3:** Effect of rootstocks type on fruit size, firmness, pH, and TSS fruits in eggplants grown in control or salt stress conditions.

Salinity	Rootstocks	Fruit Length (cm)	Fruit Width (cm)	Firmness (kg cm^−3^)	pH	Total Soluble Solids (Brix^o^)
Control	SR	16.78 ± 1.75 a,b	8.27 ± 0.96	7.75 ± 2.01	5.47 ± 0.16	2.96 ± 1.51 d
	SG	18.23 ± 0.87 a	9.21 ± 0.21	9.50 ± 2.28	5.36 ± 0.06	3.69 ± 0.95 b–d
	ST	16.58 ± 1.63 b	8.36 ± 0.77	8.98 ± 1.45	5.46 ± 0.08	4.08 ± 1.34 b–d
	SH	16.67 ± 1.77 b	8.51 ± 0.93	5.67 ± 1.10	5.51 ± 0.14	4.53 ± 1.24 b
Salinity	SR	12.45 ± 1.43 d	7.41 ± 1.06	9.44 ± 1.89	5.40 ± 0.18	4.14 ± 1.98 b,c
	SG	13.83 ± 1.04 c,d	8.16 ± 0.76	7.15 ± 0.80	5.40 ± 0.20	3.56 ± 1.20 c,d
	ST	14.30 ± 1.98 c	8.10 ± 0.84	7.51 ± 1.20	5.38 ± 0.13	5.61 ± 2.01 a
	SH	14.46 ± 1.93 c	8.43 ± 2.13	8.45 ± 1.05	5.35 ± 0.12	4.36 ± 1.45 b,c
Main effects						
Control		17.06 a	8.59 a	7.99	5.45	3.96
Salinity		13.76 b	8.02 b	8.14	5.38	4.27
	SR	14.60 b	7.84 b	8.59 a	5.44	3.85 b
	SG	16.03 a	8.69 a	8.33 a,b	5.38	3.33 b
	ST	15.41 a	8.23 a	8.25 a,b	5.42	4.84 a
	SH	15.57 a	8.47 a	7.06 b	5.43	4.44 a
Analysis of variance						
Salinity		***	*	ns	ns	ns
Rootstock		*	*	*	ns	***
Salinity× Rootstock		*	ns	ns	ns	***
CV		11.67	13.92	21.10	2.85	24.38

Different letters within the same column indicate that means ±standard deviation (four replications and 5 plants in each replication) are significantly different according to Tukey’s multiple range test at: *p* < 0.05 (*), or *p* < 0.0001 (***), ns = non-significant. —SR = self-rooted; SG = self-grafted; ST = *Solanum torvum* rootstock, SH = *Solanum grandifolium* × *Solanum melongena* rootstock.

**Table 4:** Effect of rootstocks type on fruit skin color (CIELAB color parameters) in eggplants grown in control or salt stress conditions.

Salinity	Rootstocks	L*	a*	Chroma	Hue	CIRG
Control	SR	25.41 ± 1.30	3.56 ± 1.17 c,d	3.64 ± 1.13 c,d	347.61 ± 5.77	6.53 ± 0.38 a–c
	SG	25.32 ± 0.75	3.69 ± 0.97 b–d	3.75 ± 0.94 b–d	349.66 ± 4.57	6.52 ± 0.24 b,c
	ST	25.59 ± 1.13	4.08 ± 1.30 b–d	4.13 ± 1.26 b–d	350.81 ± 5.87	6.42 ± 0.36 b,c
	SH	25.87 ± 0.95	4.53 ± 1.15 b	4.57 ± 1.12 b	337.68 ± 1.65	6.30 ± 0.29 c
Salinity	SR	24.86 ± 1.54	4.14 ± 0.90 b,c	4.20 ± 0.87 b,c	350.85 ± 4.12	6.60 ± 0.43 a,b
	SG	24.38 ± 1.12	2.96 ± 0.6 d	3.05 ± 0.56 d	346.53 ± 5.80	6.85 ± 0.35 a
	ST	25.55 ± 1.03	5.61 ± 1.24 a	5.63 ± 1.23 a	325.90 ± 3.26	6.27 ± 0.30 c
	SH	25.14 ± 1.05	4.36 ± 0.76 b,c	4.40 ± 0.74 b,c	331.01 ± 4.98	6.49 ± 0.24 b,c
Main effects						
Control		25.55 a	3.96	4.02	346.44	6.44 b
Salinity		24.98 b	4.27	4.32	338.57	6.55 a
	SR	25.13 a,b	3.85 b	3.92 b	349.23	6.57 a,b
	SG	24.85 b	3.33 b	3.40 b	348.09	6.68 a
	ST	25.57 a	4.84 a	4.88 a	338.36	6.35 c
	SH	25.50 a,b	4.44 a	4.49 a	334.34	6.40 b,c
Analysis of variance						
Salinity		**	ns	ns	ns	***
Rootstock		*	***	***	ns	*
Salinity × Rootstock		ns	***	***	ns	*
CV		4.52	24.38	24.32	15.98	5.18

Different letters within the same column indicate that means ±standard deviation (four replications and 5 plants in each replication) are significantly different according to Tukey’s multiple range test at: *p* < 0.05 (*), *p* < 0.001 (**), or *p* < 0.0001 (***), ns = non-significant. SR = self-rooted; SG = self-grafted; ST = *Solanum torvum* rootstock, SH = *Solanum grandifolium* × *Solanum melongena* rootstock.

**Table 5:** Effect of rootstocks type on flesh color (CIELAB color parameters) in eggplants grown in control or salt stress conditions.

Salinity	Rootstocks	L*_0_	Chroma	Hue	DW	CD	OP
Control	SR	87.58 ± 1.30	25.28 ± 1.69 d	101.26 ± 2.35	28.18 ± 1.92 c	8.06 ± 3.73	5.46 ± 1.63 b
	SG	87.50 ± 3.97	24.90 ± 2.63 d	100.03 ± 3.53	27.99 ± 3.58 c	8.15 ± 4.51	4.87 ± 2.12 b
	ST	85.60 ± 2.59	25.94 ± 2.19 c,d	99.69 ± 6.98	29.70 ± 3.054 b,c	9.24 ± 4.16	5.98 ± 3.59 b
	SH	84.04 ± 2.53	28.33 ± 3.31 a–c	98.44 ± 3.95	32.54 ± 3.97 ab	8.31 ± 5.31	6.05 ± 3.49 b
Salinity	SR	85.90 ± 1.77	27.61 ± 1.47 b,c	100.98 ± 3.34	31.03 ± 2.04 a–c	13.92 ± 8.27	10.18 ± 4.11 a
	SG	82.84 ± 2.78	29.69 ± 2.42 a,b	97.51 ± 4.43	31.38 ± 3.00 a–c	9.81 ± 5.02	9.24 ± 3.36 a,b
	ST	84.04 ± 3.36	26.95 ± 1.79 b–d	99.25 ± 4.63	34.35 ± 3.02 a	11.89 ± 6.30	6.84 ± 1.10 a,b
	SH	82.20 ± 2.47	30.80 ± 1.65 a	97.37 ± 5.15	35.63 ± 2.14 a	10.43 ± 7.05	6.24 ± 2.06 a,b
Main effects							
Control		86.20 a	26.12 b	99.85	29.60 b	8.44 b	5.59 b
Salinity		83.75 b	28.76 a	98.77	33.10 a	11.52 a	8.12 a
	SR	86.74 a	26.45 b	101.12 a	29.61 b	10.99	7.82
	SG	85.21 a,b	27.30 b	98.77 a,b	31.17 b	8.98	7.05
	ST	84.82 bc	26.45 b	99.47 a,b	30.54 b	10.57	6.41
	SH	83.12 c	29.56 a	97.91 b	34.08 a	9.37	6.14
Analysis of variance							
Salinity		***	***	ns	***	*	***
Rootstock		***	***	*	***	ns	ns
Salinity× Rootstock		ns	*	ns	*	ns	*
CV		2.93	8.21	4.10	9.02	3.10	21.99

Different letters within the same column indicate that means ±standard deviation (four replications and 5 plants in each replication) are significantly different according to Tukey’s multiple range test at: *p* < 0.05 (*),or *p* < 0.0001 (***), ns = non-significant. SR = self-rooted; SG = self-grafted; ST = *Solanum torvum* rootstock, SH = *Solanum grandifolium* × *Solanum melongena* rootstock. DW = whitening index, OP = oxidation potential, CD = color differences.

## Data Availability

The data presented in this study are available on request from the authors.
